# Predictors of apical periodontitis in root canal treated teeth from an adult Nepalese subpopulation: a cross-sectional study

**DOI:** 10.1186/s12903-024-04139-3

**Published:** 2024-03-29

**Authors:** Md. Asdaq Hussain, Shailendra Kumar Singh, Shazia Naz, Merazul Haque, Harish Kumar Shah, Abanish Singh

**Affiliations:** 1 Department of Conservative Dentistry & Endodontics, National Medical College, Birgunj, Nepal; 2 Department of Prosthodontics & Maxillofacial Prosthesis, National Medical College, Birgunj, Nepal; 3 Department of Operative Dentistry, de’Montmorency College of Dentistry, Lahore, Pakistan; 4 Department of Periodontology & Oral Implantology, National Medical College, Birgunj, Nepal; 5 Department of Community Dentistry, National Medical College, Birgunj, Nepal

**Keywords:** Apical periodontitis, Coronal restoration, Cross-sectional study, Periapical status, Risk factors, Root canal treatment

## Abstract

**Background:**

Endodontic literature search revealed that no study has been conducted to evaluate the prevalence of apical periodontitis (AP) in root canal treated teeth from an adult Nepalese population of Madhesh Province. Consequently, little is known about the extent and risk factors associated with it. This study aimed to determine AP prevalence in root canal treated teeth from an adult Nepalese subpopulation and to analyze the related risk factors including age, sex, tooth type, type of coronal restoration and quality of root canal treatment and coronal restoration as predictors of AP.

**Methods:**

Digital panoramic radiographs were evaluated. Periapical status of 300 root canal-treated teeth was scored by using the periapical index. The quality of root canal treatment and coronal restorations were categorized as adequate or inadequate through radiographic and clinical evaluation. The data were analyzed using univariate and multivariate logistic regression models.

**Results:**

Prevalence of AP in the present study was 31.7%. In 45.7% of the treated teeth, quality of root canal treatment was adequate whereas 46% of the cases had adequate coronal restorations. Multivariate logistic regression analysis revealed statistically significant associations and remarkably increased risk for AP in teeth with inadequate root canal treatment (odds ratio [OR] = 7.92; 95% CI: 3.96–15.82; *p* < 0.001) whereas lower risk for AP was found in females (OR = 0.51; 95% CI: 0.28–0.90; *p* = 0.021) and in teeth restored with crown (OR = 0.22; 95% CI: 0.09–0.51; *p* < 0.001) and filling (OR = 0.18; 95% CI: 0.08–0.42; *p* < 0.001). Quality of coronal restoration, tooth type and age of the patient were not found to be the predictors of AP.

**Conclusions:**

Within the limits of this study, a high prevalence of AP and poor overall quality of root canal treatment and coronal restoration was found in the subpopulation studied. Quality of root canal treatment, type of coronal restoration and sex of the patient are significant predictors of possible AP development in root canal treated teeth. Substantial efforts are needed to improve the endodontic treatment standards.

**Supplementary Information:**

The online version contains supplementary material available at 10.1186/s12903-024-04139-3.

## Background

The oral cavity consists of tissues and organs that form an integral part of the stomatognathic system [1]. Many infectious and inflammatory diseases of the oral cavity have been associated with systemic diseases [2]. Dental caries and periodontitis are two of the most common oral diseases that present a significant public health problem and result in a high economic burden [3]. Apical periodontitis (AP) is an inflammatory disease [4] which in most cases is a direct sequela of dental caries [5]. Caries leads to necrosis of pulpal tissue and continual spread of infection in the periapical region [6]. For diagnosis of AP, radiographic evaluation is important since AP can develop and continue without detectable clinical signs [4].

Root canal treatment (RCT) is indicated for irreversible pulpitis and/or apical periodontitis [7]. The objective of RCT is to prevent and treat AP by eliminating microorganisms and necrotic pulp through chemomechanical debridement and providing an adequate root filling so as to seal the canals and prevent reinfection [8]. Contamination of root canal may occur in teeth with or without previous root canal treatment [9]. A systematic review with meta-analysis showed a very high AP prevalence (39%) in root treated teeth compared to nontreated teeth (3%) [10].

A cross sectional study has been shown to be the best measure of the status of health, disease and therapy in a population [11]. In developing countries, reports on oral diseases are limited. Thus, the basis for the scope of oral health schemes may be the study carried out in different countries which does not correctly signify the situations of developing countries [12]. Taking into account the high prevalence of AP globally [6], the unequal distribution of oral diseases across populations [3] and RCT as one of the most frequent treatments performed worldwide [13], it is reasonable to evaluate the epidemiology of AP in root canal treated teeth from Nepalese population. This will help in the identification and treatment of patients or teeth having higher probability of developing AP. Moreover, it will enable the purposeful distribution of resources, arrangement, and drafting of dental, specifically endodontic, workforce and training [4].

Endodontic literature search revealed that no study has been conducted to evaluate the prevalence of apical periodontitis (AP) in root canal treated teeth from an adult Nepalese population of Madhesh Province. Consequently, little is known about the extent and risk factors associated with it. The aim of the present study was to determine AP prevalence in root canal treated teeth from an adult Nepalese subpopulation and to analyze the related risk factors including age, sex, tooth type, type of coronal restoration and quality of root canal treatment and coronal restoration as predictors of AP.

## Methods

This cross-sectional study was carried out on patients attending the dental outpatient department (OPD) of National Medical College & Teaching Hospital (NMCTH), Birgunj, Nepal for the first time seeking routine dental care between December 2022 and May 2023. Ethical clearance for the study was obtained from the Institutional Review Committee of NMCTH Birgunj (Ref. F-NMC/613/079–080). Informed consent was obtained from all subjects and/or their legal guardian(s) prior to inclusion in the study. This study was reported in compliance with the “strengthening the reporting of observational studies in epidemiology” (STROBE statement) [14].

### Patient selection

The inclusion criteria of the study were as follows:

- Adult patients ≥ 18 years of age.
- Patients with full complementary natural teeth or a minimum of 10 remaining natural teeth.
- Patients undergoing orthopantomography (OPG) for diagnosis and treatment plan.
- Patients with ≥ 1 root canal treated tooth. RCTs were performed either at NMCTH, Birgunj or other centers in Madhesh Province.

The exclusion criteria of the study were as follows:

- Medically compromised patients.
- Root-treated teeth that have undergone endodontic surgery i.e., root resections or hemi-section.
- Third molars teeth.
- Immature teeth.
- Teeth that could not be assessed radiographically due to superimposition of anatomic structures.

The sample size of the study was calculated based on standard formula [15], considering 85% power at a 95% confidence interval (CI).

$$n = \frac{{{Z^2}{\rm{P}}(1 - {\rm{P}})}}{{{d^2}}}$$

With reference to the findings of a published study [16], the prevalence (P) of AP was evaluated at 23.8%. Using the above formula, the sample size estimated was 279 teeth. Considering that 5% of the OPGs could not be evaluated because of inadequate quality, the final sample size taken was 300 teeth.

### Clinical examination

Clinical examination was carried out by one examiner (MAH). Any ambiguous situations were further discussed with two other observers and a consensus was reached. The clinical examination procedures given by the American Association of Endodontics [17] were followed which included parameters such as the presence of periodontal pockets, sinus tract formation, tooth mobility, facial symmetry, tenderness on percussion and palpation of adjacent soft tissues. The type of coronal restoration (CR) was recorded. Direct restoration i.e., a direct chairside amalgam or composite filling were categorized as filling. Indirect restoration i.e., a ceramic or a metal-ceramic crown, fabricated in a laboratory and then cemented were categorized as crown. The quality of coronal restorations was evaluated clinically and radiographically in terms of anatomical form, marginal integrity and presence of recurrent caries using the simplified form of the Modified Ryge’s criteria [18].

### Radiographic examination

Digital orthopantomograms (OPGs) of all the participants were taken at the Department of Oral Radiology, National Medical College, Birgunj by a well-trained dental radiographer using a panoramic digital radiography device PaX-i 2D Imaging System (VATECH Global, Korea, Model: PCH-2500). All the digital images were assessed by a single examiner (MAH), who is an endodontist. For assessment of the radiographs, an 18.5-inch liquid crystal monitor (E1912H; Dell, Austin, Texas, United States; resolution: 1280 × 1024 64-bit color; graphic card: HD Graphics; Intel, Santa Clara, California, United States) using EzDent-i software (VATECH Global, Korea) was used. Software image enhancement functions were used by the examiner whenever the need was felt for better assessment. Before the study, 20 OPGs, of which 20 reference root canal-treated teeth not included in this cross-sectional evaluation were used for calibration of the single examiner. Intra-examiner agreement for the periapical index scores in root canal-treated teeth was measured by computing Cohen’s Kappa coefficient (κ) after re-examining the reference teeth. The κ value for intra-examiner agreement was 0.78 indicating substantial agreement.

### Root canal treatment evaluation

Teeth obturated with a radio-opaque material in the pulp chamber and/or in one or more of the root canals were categorized as root canal treated. In multirooted teeth, the canal with the most incomplete filling was taken into consideration. The evaluation criteria described previously by Tronstad et al. [19] and Tavares et al. [20] were used in slightly modified form, which states:

- Adequate treatment: All canals obturated. Voids not present. Root canal fillings end from 0 to 2 mm short of the radiographic apex.
- Inadequate treatment: Grossly deficient root fillings (Root canal fillings end > 2 mm short of the radiographic apex or with inadequate density, unfilled canals, and/or poor compaction) or grossly overfilled.

### Coronal restoration evaluation

The coronal restoration quality was determined both clinically and radiographically. The evaluation criteria used were somewhat modified from those stated by Tronstad et al. [19] and Song et al. [21] as follows:

- Adequate restoration: Any permanent restoration that seemed intact radiographically and had no comment on the clinical examination record.
- Inadequate restoration: Any permanent restoration with detectable radiographic signs of overhangs, recurrent caries, open margins or presence of temporary coronal restoration or comments such as “ill-fitting margin” or “secondary dental caries” on the clinical examination record. Teeth with no coronal restorations, permanent or temporary, were also included in this group.

### Periapical status evaluation

The Periapical Index (PAI) scoring system [22] was used to assess the presence or absence of AP in each sample tooth, using a scale ranging from 1 to 5. Healthy teeth were designated by scores of 1 or 2 while scores of 3 to 5 specified the presence of AP. Multirooted teeth were categorized according to the root with the worst score. The five categories used were as follows:

1. Normal periapical structures.
2. Small changes in periapical bone structure.
3. Changes in periapical bone structure along with some mineral loss.
4. Periodontitis with a well-defined radiolucent area.
5. Severe periodontitis with elements showing expansion of the lesion.

All teeth were categorized according to the Fédération Dentaire Internationale (FDI) nomenclature. The data recorded on a structured proforma for each patient were: age and sex; teeth present (excluding third molars); type of teeth that were root canal treated: incisors, canines, premolars or molars; type of coronal restorations in root treated teeth: an indirect restoration i.e., full coverage crown or a direct restoration i.e., filling; quality of RCT, quality of CR, and the PAI score of the root-treated teeth.

### Statistical analysis

Raw data obtained were entered and coded in Excel sheets (Microsoft; Redmond, WA, USA). All statistical analyses were performed by using SPSS software (Statistical Package for Social Sciences, version 24.0, IBM, Chicago, IL, USA). Risk of AP was evaluated by means of odds ratios (OR) and the corresponding 95% confidence interval. A *p*-value of < 0.05 was considered statistically significant. The outcome variable was the presence versus absence of AP in the tooth. Univariate logistic regression model was used to investigate the association of diverse variables with AP. The variables having significant associations in univariate analysis were entered in multivariate regression model to find significant predictors of AP.

## Results

A total of 172 patients were selected in the present study, of whom 74 were males and 98 were females. The average patient age was 41.55 years (range = 18–80 years). From the selected patients, a total number of 300 root canal treated teeth were assessed. Table 1 shows a descriptive analysis of the study sample. Of the 300 teeth, 128 (42.7%) were from males and 172 (57.3%) were from females. The highest prevalence of root treated teeth was in the 18–29 years age category (22.7%) followed by the ≥ 60 years age category (21.7%). Among the types of teeth, molars were the most commonly root canal treated (54.3%). On the basis of PAI scoring criteria, 205 (68.3%) teeth were categorized as healthy and 95 (31.7%) teeth had AP.

**Table 1:** Description of the study population (*N* = 300)

Variables	Categories	Frequency (*N*)	Percentage (%)
Age Category (years)	18–29	68	22.7
	30–39	61	20.3
	40–49	51	17.0
	50–59	55	18.3
	> 60	65	21.7
Sex	Male	128	42.7
	Female	172	57.3
Tooth Type	Incisors	48	16.0
	Canines	14	4.7
	Premolars	75	25.0
	Molars	163	54.3
Root Canal Treatment	Adequate	137	45.7
	Inadequate	163	54.3
Coronal Restoration	Adequate	138	46.0
	Inadequate	162	54.0
Coronal Restoration Type	Crown	126	42.0
	Filling	128	42.7
	Lost Restoration	46	15.3
Periapical Index Score	1	129	43.0
	2	76	25.3
	3	62	20.7
	4	24	8.0
	5	9	3.0
Periapical Status	Healthy	205	68.3
	Apical periodontitis	95	31.7

(*N* = 300): number of sample teeth

Table 2 presents the descriptive data of root canal treatment and coronal restoration quality. Short obturation i.e., grossly deficient root fillings, was found in 38.3% of the sample. Coronal restorations with recurrent caries were seen in 19% of root treated teeth.

**Table 2:** Descriptive data of root canal treatment and coronal restoration quality (*N* = 300)

Variables	Condition	Frequency (*N*)	Percentage (%)
Root Canal Treatment Quality	Adequate	137	45.7
	Inadequate density	2	0.7
	Grossly overfilled	9	3.0
	Poor condensation	12	4.0
	> 2 mm short	115	38.3
	Unfilled canals	25	8.3
Coronal Restoration Quality	Adequate	138	46.0
	Lost restoration	50	16.7
	Open margins	15	5.0
	Overhangs	34	11.3
	Recurrent caries	57	19
	Temporary restoration	6	2.0

Table 3 depicts the results of unadjusted (univariable) logistic regression model for diverse variables association with AP. A decreased risk for AP was demonstrated in females (OR = 0.51; 95% CI: 0.31–0.84; *p* = 0.009) than males. In contrast, the association between the variables age category and tooth type with AP was not apparent. The number of teeth with adequate root canal therapy was 137 (45.7%). Of these adequately treated teeth, 89.8% were classified as healthy (AP absent) and 10.2% were classified as diseased (AP present). The number of root fillings ranked as inadequate was 167 (54.3%). Out of these teeth with inadequate treatment, 50.3% were scored as healthy and 49.7% had AP. The teeth with inadequate root canal treatment showed an increased AP risk (OR = 8.67; 95% CI: 4.61–16.33; *p* < 0.001).

**Table 3:** Univariate logistic regression for association of diverse variables with apical periodontitis

Variables	Total *N* (%)	Periapical Status		β	OR	CI at 95%	*p*-value
		AP absent *n* (%)	AP present *n* (%)				
Age Category (Years)							
18–29	68 (22.7)	51 (75.0)	17 (25.0)		1	Reference	
30–39	61 (20.3)	43 (70.7)	18 (29.5)	0.22	1.25	0.57–2.73	0.566
40–49	51 (17.0)	27 (52.9)	24 (47.0)	0.98	2.66	1.22–5.79	0.013
50–59	55 (18.3)	40 (72.7)	15 (27.3)	0.11	1.12	0.50–2.52	0.775
≥ 60	65 (21.7)	44 (67.7)	21 (32.3)	0.35	1.43	0.67–3.04	0.352
Sex							
Male	128 (42.7)	77 (60.1)	51 (39.8)		1	Reference	
Female	172 (57.3)	128 (74.4)	44 (25.6)	-0.65	0.51	0.31–0.84	0.009*
Tooth Type							
Incisors	48 (16.0)	38 (79.1)	10 (20.9)		1	Reference	
Canines	14 (4.7)	10 (71.4)	4 (28.5)	0.41	1.52	0.39–5.87	0.544
Premolars	75 (25.0)	53 (70.7)	22 (29.3)	0.45	1.57	0.67–3.71	0.297
Molars	163 (54.3)	104 (63.8)	59 (36.2)	0.76	2.15	1.002–4.63	0.049
Root Canal Treatment Quality							
Adequate	137 (45.7)	123 (89.8)	14 (10.2)		1	Reference	
Inadequate	163 (54.3)	82 (50.3)	81 (49.7)	2.16	8.67	4.61–16.33	< 0.001*
Coronal Restoration Quality							
Adequate	138 (46.0)	117 (84.8)	21 (15.2)		1	Reference	
Inadequate	162 (54.0)	88 (54.3)	74 (45.7)	1.54	4.68	2.68–8.18	< 0.001*
Coronal Restoration Type							
Lost Restoration	46 (15.3)	17 (36.9)	29 (63.1)		1	Reference	
Crown	126 (42.0)	93 (73.8)	33 (26.2)	-1.57	0.20	0.10–0.42	< 0.001*
Filling	128 (42.7)	95 (74.2)	33 (25.8)	-1.59	0.20	0.09–0.41	< 0.001*

Abbreviations: AP, apical periodontitis; CI, confidence interval; OR, odds ratio. *Statistically significant

Forty-six percent of the root canal treated teeth comprised in this study presented coronal restoration classified as adequate. Of these, 84.8% were ranked as healthy. Inadequate coronal restorations were found in 54% of the teeth. Of these, only 54.3% were scored as healthy. A significant association with presence of AP was seen in teeth with inadequate quality restorations (OR = 4.68, 95% CI: 2.68–8.18; *p* < 0.001; Table 3). Regarding the type of coronal restorations, 42.0% of the endodontically treated teeth were restored with crown and 42.7% with filling, whereas 15.3% had no coronal restorations i.e., lost restorations. Taking lost restoration as a reference category, statistically significant association and decreased AP risk was demonstrated in teeth restored with crown and filling, (OR = 0.20; 95% CI: 0.10–0.42; *p* < 0.001) and (OR = 0.20; 95% CI: 0.09–0.41; *p* < 0.001), respectively.

Univariate logistic regression was also applied to analyse the association of combined quality of root canal treatment and coronal restoration with AP (Table 4). The combination of adequate RCT and adequate CR showed the lowest AP prevalence (2.2%) and this combination was taken as a reference category. Statistically significant associations and increased AP risk was seen in teeth with adequate RCT and inadequate CR (OR = 16.52; 95% CI: 3.54–77.03; *p* < 0.001), teeth with inadequate RCT and adequate CR (OR = 28.60; 95% CI: 6.24-130.97; *p* < 0.001) and teeth with inadequate RCT and inadequate CR (OR = 52.05; 95% CI: 12.22–221.60; *p* < 0.001).

**Table 4:** Univariate logistic regression for association of combined quality of root canal treatment and coronal restoration with apical periodontitis

Covariates	Total *N* (%)	Periapical Status		β	OR	CI at 95%	*p*-value
		AP absent *n* (%)	AP present *n* (%)				
Adequate RCT/ Adequate CR	91 (30.3)	89 (97.8)	2 (2.2)		1	Reference	
Adequate RCT/ Inadequate CR	48 (16.0)	35 (72.9)	13 (27.1)	2.80	16.52	3.54–77.03	< 0.001*
Inadequate RCT/ Adequate CR	46 (15.3)	28 (60.8)	18 (39.2)	3.35	28.60	6.24-130.97	< 0.001*
Inadequate RCT/ Inadequate CR	115 (38.3)	53 (46.0)	62 (53.9)	3.95	52.05	12.22–221.60	< 0.001*

Abbreviations: AP, apical periodontitis; RCT, root canal treatment; CR, coronal restoration; CI, confidence interval; OR, odds ratio. *Statistically significant

In multivariate model, only those variables having significant associations in univariate regression analysis, were included (Table 5). The results showed that three variables i.e., sex, root canal treatment quality and coronal restoration type maintained the significant associations with AP presence. Females were less susceptible to AP development in root canal treated teeth in contrast to males (OR = 0.53; 95% CI: 0.30–0.95; *p* = 0.035). Regarding root canal treatment quality, the OR for the AP presence was 7.92 times higher if RCT quality was inadequate (OR = 7.92; 95% CI: 3.96–15.82; *p* < 0.001). When comparing to the lost restorations, root canal treated teeth restored with crown and filling showed significantly decreased odds for AP presence, (OR = 0.30; 95% CI: 0.12–0.71; *p* = 0.007) and (OR = 0.25; 95% CI: 0.10–0.59; *p* = 0.002), respectively.

**Table 5:** Multivariate logistic regression for association of diverse variables with apical periodontitis

Variables	Total *N* (%)	Periapical Status		β	OR	CI at 95%	*p*-value
		AP absent *n* (%)	AP present *n* (%)				
Sex							
Male	128 (42.7)	77 (60.1)	51 (39.8)		1	Reference	
Female	172 (57.3)	128 (74.4)	44 (25.6)	-0.61	0.53	0.30–0.95	0.035*
Root Canal Treatment Quality							
Adequate	137 (45.7)	123 (89.8)	14 (10.2)		1	Reference	
Inadequate	163 (54.3)	82 (50.3)	81 (49.7)	2.07	7.92	3.96–15.82	< 0.001*
Coronal Restoration Quality							
Adequate	138 (46.0)	117 (84.8)	21 (15.2)		1	Reference	
Inadequate	162 (54.0)	88 (54.3)	74 (45.7)	0.63	1.89	0.98–3.65	0.057
Coronal Restoration Type							
Lost Restoration	46 (15.3)	17 (36.9)	29 (63.1)		1	Reference	
Crown	126 (42.0)	93 (73.8)	33 (26.2)	-1.19	0.30	0.12–0.71	0.007*
Filling	128 (42.7)	95 (74.2)	33 (25.8)	-1.38	0.25	0.10–0.59	0.002*

Abbreviations: AP, apical periodontitis; CI, confidence interval; OR, odds ratio. *Statistically significant

## Discussion

The current study aimed to evaluate the prevalence and determining factors of AP in root canal treated teeth from an adult Nepalese subpopulation of Madhesh Province. The findings of this study demonstrate that the AP prevalence in root treated teeth was 31.7%, which is comparable to earlier studies that used panoramic radiographs [23, 24]. A systematic review with meta-analysis [11] showed AP presence in 36% of cases. High AP prevalence observed in this study was mostly associated to the higher prevalence of teeth with inadequate root canal therapy.

This study marks the inaugural research effort of its kind conducted in the Madhesh Province. The sample examined consisted of patients who visited dental OPD of NMCTH Birgunj, situated in the Madhesh Province of Nepal. Similar screening criteria based on dental school patient selection have been employed in several other prevalence studies [16, 21, 24]. As NMCTH Birgunj stands as the sole dental institute and tertiary care referral dental center in Madhesh Province, individuals from all districts of the province avail themselves of its specialized dental services. Madhesh Province, located in the southeastern Terai region of Nepal and bordering Koshi Pradesh to the east and north, Bagmati Province to the north, and India’s Bihar state to the south and west, exhibits a population distribution of 50.1% males and 49.9% females [25]. Nonetheless, it is crucial to note that our subjects did not constitute a random sample of the Madhesh province population; rather, they were individuals actively seeking dental treatment. Therefore, the results are limited to the specific group studied and may not be applicable to the general population. However, in this study, the AP frequency and its associations with variables can be identified.

Based on the existing data, Nepal has a dentist-to-population ratio of 1:20000, nearly three times below the World Health Organization’s (WHO) recommended ratio [26]. The majority of practitioners in the province are general dentists, and the distribution of specialization in subdisciplines is limited. Regrettably, instances of quackery and fraudulent dental practices are noted within the province [26].

In the present study, panoramic radiograph was used to score the periapical status. Several epidemiological studies have used dental panoramic radiographs for evaluating the AP and the technical quality of RCT [16, 24]. Panoramic radiographs were employed in this study due to their availability, given that full-mouth periapical radiographs were not routinely taken in our college. With panoramic images, all teeth present can be visualized on one radiograph and a large amount of data is comfortably accumulated without exposing the individuals to extra radiation. These features make panoramic radiographs beneficial compared to full-mouth periapical radiographs [24]. Additionally, in the identification of periapical lesions, no significant difference has been found between the sensitivities of panoramic and full-mouth periapical radiographs [27, 28]. Contemporary panoramic machines generate higher-quality radiographic images, including improved clarity in the anterior region. Consequently, panoramic radiographs are considered a suitable diagnostic tool for assessing apical lesions in epidemiological studies related to dental care [27, 28].

Our study sample consisted of more females (56.97%) than males (43.02%), which may reflect the considerable interest of females within Nepal in receiving dental treatment. The predominance of female patients has also been shown in similar epidemiologic studies; however, those studies reported that gender did not correlate with the presence of AP [29, 30]. According to our results, females are less likely to have AP development in root canal-treated teeth than males. It is difficult to clarify the observed gender difference in our study. It would make sense if the males underwent root canal treatment when their teeth were in worse condition than females, potentially resulting in a poorer prognosis for therapy in males. Unfortunately, in this study, the preoperative condition, as well as details about the individuals who performed endodontic treatment, were not known. Future studies could help in understanding gender differences in the development of periapical lesions in root-filled teeth. In alignment with our study, Bürklein et al. [31] also observed a gender difference and linked it to diverse biopsychosocial factors affecting pain perception, including genetic factors, endogenous opioid function, sex hormones, pain coping, and gender roles. They further added that women tend to be more pain-sensitive and are more likely to seek dental care [31]. Corroborating our findings on gender differences, Jakovljevic et al. [6], in a meta-analysis, emphasized that despite conflicting outcomes in primary studies regarding the role of gender as a predisposing factor for the development of AP, numerous investigations have highlighted significant variations in oral hygiene practices between males and females.

More than half of the cases evaluated in the present study had inadequate root canal treatment. Similar findings have been observed in earlier studies conducted on different populations [32–34]. When analysing the causes of inadequate quality of RCTs, it was found that the most common cause was short obturation. This finding is consistent with previous studies [35, 36]. According to Strindberg [37], the success rate of endodontic treatment was highest when the root canal filling length was 1 mm shorter than the apex. The existence of microorganisms in the non-instrumented portions of the root canal may have caused the failure in teeth having short root fillings [38].

Previous studies have shown a lower apical disease rate in teeth with adequately filled root canals [19, 39]. Similar findings were observed in this study where 89.8% of adequate root treated teeth showed absence of AP. Furthermore, consistent with the earlier findings [21, 24], adequately root canal-treated teeth had significantly better periapical health than inadequately treated teeth. According to Siqueir Jr et al. [38], the major aetiological factor of post-treatment AP is persistent intra-radicular infection resulting from inadequate treatment which is similar to the finding of this study. Actually, multivariate logistic regression analysis demonstrated that the quality of root canal therapy was the most significant predictor of AP in root treated teeth. Compared to the teeth with adequate root canal treatment, AP was present approximately eight times more frequently in teeth with inadequate treatment. On the other hand, few cross-sectional studies [40, 41] have revealed no significant relationship between the quality of root canal therapy and the AP presence.

10% of teeth, despite having adequate quality RCTs, still had AP in the current study. This shows that the RCT quality is not the solitary factor affecting the periapical health of endodontically treated teeth. As stated by Moreno et al. [42], the coronal restoration quality is another factor that may affect the apical status. Similar to their findings [42], only 46% of the coronal restorations were classified as adequate in this study. The most common cause of inadequate quality restorations was recurrent caries. This emphasizes the significance of an adequate coronal seal after endodontic treatment. In univariate analysis, significantly better periapical health was found in teeth with adequate coronal restorations compared to the inadequate restorations. This result is analogous with the studies performed in Kuwait [43] and Jordan [29]. However, corresponding to the findings of Bukmir et al. [30], when the multivariate logistic regression was applied, this relationship did not remain significant. The presence of other significant variables in the multivariate model may have influenced this outcome.

In this study, a significant association was found between the type of coronal restoration and presence of AP. This is in agreement with the study by Frisk et al. [44]. This association may be due to the microleakage occurring from poor marginal integrity of the restorations which could not be identified clinically [44]. Moreover, other determining factors of AP might also have influenced the periapical status of the root-treated teeth. Contrary to this association, El Ouarti [45] found no relationship between coronal restoration type and AP.

Combined data for coronal restoration and root canal treatment quality yielded noticeable results. When both coronal restoration and root canal treatment were adequate, only 2.2% of the treated teeth had AP, however if both were inadequate, 53.91% of the teeth had AP. This strongly denotes that both factors i.e., quality of endodontic treatment and quality of coronal restoration play important roles in maintaining the periapical health of root treated teeth. Significantly worse outcome and remarkably increased odds for AP was found when either root canal treatment or coronal restoration was inadequate or when both were inadequate. The meta-analysis conducted by Gillen et al. [46] validated this finding, which showed that the odds for healing capacity of AP increase in teeth with both adequate endodontic treatment and adequate coronal restoration.

The main limitation of the present study was the usage of OPGs for assessing the periapical status. Disadvantages of OPGs are well known including a two-dimensional image of three-dimensional structures, superimposition, anatomic noise and geometric distortion. Furthermore, to be radiographically apparent, apical radiolucency should reach approximately 30-50% of the bone mineral loss. Thus, AP might be existing though not identified radiographically [47]. Cone beam computed tomography (CBCT) surpasses panoramic and periapical radiographs, as three-dimensional view permits more accurate estimation of the size and location of the lesion in addition to the detection of lateral voids and missed canals in the evaluation of technical quality [48]. But all dental practices are not equipped with CBCT [49]. More notably, owing to augmented risk of radiation, the CBCT indications are highly regulated, mostly limited to the zone of interest instead of the complete dentition [50].

Other limitations of this study were the lack of information concerning the evaluated cases which may affect the root canal treatment outcome, for example the operator factor, whether trained or untrained doctor performed the treatment in single or multiple visits and whether permanent restoration was placed just after root canal treatment completion. Furthermore, there was no information about the absence or presence of periapical radiolucency before root canal treatment (no preoperative radiograph was available), elapsed time of endodontic treatment, microbiological environment of the root canal, periodontal status and oral hygiene habits of the patients. Further prospective controlled trials are recommended to evaluate the detail role of each factor responsible for AP, clinically and radiographically.

## Conclusions

Within the limits of this study, a high prevalence of AP and poor overall quality of root canal treatment and coronal restoration was found in the subpopulation studied. Quality of root canal treatment, type of coronal restoration and sex of the patient are significant predictors of possible AP development in root canal treated teeth. Substantial efforts are needed to improve the endodontic treatment standards.

### Electronic supplementary material

Below is the link to the electronic supplementary material.

Supplementary Material 1

## Data Availability

The datasets used during the present study are available from the corresponding author on reasonable request.

## Abbreviations

AP: Apical periodontitis
RCT: Root canal treatment
CR: Coronal restoration
OPG: Orthopantomogram
PAI: Periapical index
SPSS: Statistical package for social sciences
OR: Odds ratio
FDI: Fédération Dentaire Internationale
NMCTH: National Medical College & Teaching Hospital
STROBE: Strengthening the reporting of observational studies in epidemiology
CI: Confidence interval
CBCT: Cone-beam computed tomography
WHO: World Health Organization
