# Supplementary material for: Predictors of apical periodontitis in root canal treated teeth from an adult Nepalese subpopulation: a cross-sectional study
Source: BMC Oral Health. 2024 Mar 29;24:400. doi: 10.1186/s12903-024-04139-3 (PMC10979567; doi:10.1186/s12903-024-04139-3)
Supplement: Supplementary file 1 — Supplementary Material 1 [file 12903_2024_4139_MOESM1_ESM.docx]

STROBE Statement—Checklist of items that should be included in reports of ***cross-sectional studies***

|  | Item No | Recommendation |  |
| --- | --- | --- | --- |
| **Title and abstract** | 1 | (*a*) Indicate the study’s design with a commonly used term in the title or the abstract | Page 2: Abstract Paragraph 2 |
|  |  | (*b*) Provide in the abstract an informative and balanced summary of what was done and what was found | Page 3: Abstract Paragraph 2, 3 & 4 |
| Introduction | | |  |
| Background/rationale | 2 | Explain the scientific background and rationale for the investigation being reported | Page 4 Background:  Paragraph 1 & 2  Page 4 & 5 Background:  Paragraph 3 & 4 |
| Objectives | 3 | State specific objectives, including any prespecified hypotheses | Page 5 Background: Paragraph 4; Hypotheses: NA |
| Methods | | |  |
| Study design | 4 | Present key elements of study design early in the paper | Page 5 Methods: Paragraph 1 |
| Setting | 5 | Describe the setting, locations, and relevant dates, including periods of recruitment, exposure, follow-up, and data collection | Page 5 Methods: Paragraph 1 |
| Participants | 6 | (*a*) Give the eligibility criteria, and the sources and methods of selection of participants | Page 5 & 6 Methods: Paragraph 1, 2 & 3 |
| Variables | 7 | Clearly define all outcomes, exposures, predictors, potential confounders, and effect modifiers. Give diagnostic criteria, if applicable | Page 6, 7, 8 & 9 Methods: Paragraph 5, 6, 7, 8, 9 & 10 |
| Data sources/ measurement | 8 | For each variable of interest, give sources of data and details of methods of assessment (measurement). Describe comparability of assessment methods if there is more than one group | Page 6, 7, 8 & 9 Methods: Paragraph 5, 6, 7, 8, 9 & 10 |
| Bias | 9 | Describe any efforts to address potential sources of bias | Page 6 & 7 Methods: Paragraph 5 & 6 |
| Study size | 10 | Explain how the study size was arrived at | Page 6 Methods: Paragraph 4 |
| Quantitative variables | 11 | Explain how quantitative variables were handled in the analyses. If applicable, describe which groupings were chosen and why | NA |
| Statistical methods | 12 | (*a*) Describe all statistical methods, including those used to control for confounding | Page 9 Methods: Paragraph 11 |
|  |  | (*b*) Describe any methods used to examine subgroups and interactions | NA |
|  |  | (*c*) Explain how missing data were addressed | NA |
|  |  | (*d*) If applicable, describe analytical methods taking account of sampling strategy | NA |
|  |  | (*e*) Describe any sensitivity analyses | Page 7 Methods: Paragraph 6 |
| Results | | |  |
| Participants | 13 | (a) Report numbers of individuals at each stage of study—eg numbers potentially eligible, examined for eligibility, confirmed eligible, included in the study, completing follow-up, and analysed | Page 9 Results: Paragraph 1 |
|  |  | (b) Give reasons for non-participation at each stage | NA |
|  |  | (c) Consider use of a flow diagram | NA |
| Descriptive data | 14 | (a) Give characteristics of study participants (eg demographic, clinical, social) and information on exposures and potential confounders | Page 9 Results: Paragraph 1 |
|  |  | (b) Indicate number of participants with missing data for each variable of interest | NA |
| Outcome data | 15 | Report numbers of outcome events or summary measures | Page 9, 10, 11, 12,13, 14 Results: Table 1, 2, 3, 4, 5 |
| Main results | 16 | (*a*) Give unadjusted estimates and, if applicable, confounder-adjusted estimates and their precision (eg, 95% confidence interval). Make clear which confounders were adjusted for and why they were included | Page 9, 10, 11, 12, 13, 14 Results: Paragraph 1, 2, 3, 4, 5, 6 |
|  |  | (*b*) Report category boundaries when continuous variables were categorized | Page 9, 10 Results: Table 1 |
|  |  | (*c*) If relevant, consider translating estimates of relative risk into absolute risk for a meaningful time period | NA |
| Other analyses | 17 | Report other analyses done—eg analyses of subgroups and interactions, and sensitivity analyses | NA |
| Discussion | | |  |
| Key results | 18 | Summarise key results with reference to study objectives | Page 15, 16 & 17 Discussion Paragraph 1, 3, 4, 5, 6, 7 & 8 |
| Limitations | 19 | Discuss limitations of the study, taking into account sources of potential bias or imprecision. Discuss both direction and magnitude of any potential bias | Page 18 Discussion Paragraph 10 & 11 |
| Interpretation | 20 | Give a cautious overall interpretation of results considering objectives, limitations, multiplicity of analyses, results from similar studies, and other relevant evidence | Page 15,16, 17 & 18 Discussion Paragraph 1, 2,3, 4, 5, 6, 7, 8, 9, 10 & 11 |
| Generalisability | 21 | Discuss the generalisability (external validity) of the study results | Page 17 Discussion Paragraph 9 |
| Other information | | |  |
| Funding | 22 | Give the source of funding and the role of the funders for the present study and, if applicable, for the original study on which the present article is based | Page 19 Declarations, Funding |
